# Evaluation of storage stability of soybean biodiesel using a flame ionization detector coupled to a gas chromatography system

**DOI:** 10.1186/1753-6561-8-S4-P210

**Published:** 2014-10-01

**Authors:** Mozart D Bispo, Flávia Manuella R de Mendonça, Jamilly AS Barros, Cláudio Dariva, Laiza C Krause

**Affiliations:** 1 Pós-Graduação em Biotecnologia Industrial, Universidade Tiradentes, Aracaju, SE, Brazil; 2 Curso de Farmácia, Universidade Tiradentes, Aracaju, SE, Brazil; 3 Pós-Graduação em Engenharia de Processos, Universidade Tiradentes, Aracaju, SE, Brazil

## Background

Biodiesel, an alternative energy source, has been attracting increasing worldwide interest in recent decades. It is obtained by base-catalyzed transesterification, resulting in a final mixture consisting of mono-alkyl esters and other by-products (*e.g*. glycerol, alcohol, free fatty acids). This composition provides a level of operational quality control that enables biodiesel to be marketed efficiently, because its viability depends on several factors, such as stability during the storage period. Unlike fossil fuels, which are relatively inert and therefore undergo few alterations to their properties during storage, biodiesel degrades through oxidation and hydrolysis, with consequent alterations to its properties, due to exposure to the environment. Thus, the purpose of this study was to monitor soybean biodiesel quality using a flame ionization detector (FID) coupled to a gas chromatography (GC) system.

## Methods

In the alkali catalytic methanol transesterification method, the catalyst (KOH) is dissolved in methanol by vigorous stirring in a small reactor. The oil is transferred into the biodiesel reactor, and then, the catalyst/alcohol mixture is pumped into the oil. A successful transesterification reaction produces two liquid phases: ester and crude glycerin. Crude glycerin, the heavier liquid, will collect at the bottom after several hours of settling. Phase separation can be observed within 10 min and can be complete within 2 h of settling. Soybean biodiesel samples were stored for 7, 15 and 30 days at two different temperatures (room temperature and 8°C). GC-FID was used as single methodology to evaluate the ester content and storage stability of the samples in the following manner: a DB-Waxetr capillary column (30m×0.25mm, 0.25*μ*m), injection volume of 1*μ*L, and N_2 _as carrier gas. The column temperature was maintained at 170°C for 1min, and then ramped up at 10°Cmin^−1 ^to 210°C, kept at 210°C for 1min, then ramped up at 5°Cmin^−1 ^to 230°C, and kept at 230°C for 6min. These samples were then compared to standard compounds. The FID temperature was 230°C.

## Results and conclusion

The GC-FID analysis showed results that allowed biodiesel esters to be identified and quantified, indicating biodiesel degradation during storage periods, a decrease in ester content of 14.40% and 16.0% at 8°C and room temperature, respectively. The GC-FID method is especially suitable as a rapid tool for control purposes in order to determine the methyl ester content quickly, simply and inexpensively, in order to meet Brazil's National Agency of Petroleum, Natural Gas and Biofuels (ANP) requirements for biodiesel commercialization.
